# MUC3A promotes non-small cell lung cancer progression via activating the NFκB pathway and attenuates radiosensitivity

**DOI:** 10.7150/ijbs.59430

**Published:** 2021-06-16

**Authors:** Yingming Sun, Xiaoge Sun, Chengcheng You, Shijing Ma, Yuan Luo, Shan Peng, Fang Tang, Xiaoli Tian, Feng Wang, Zhengrong Huang, Hongnv Yu, Yu Xiao, Xiaoyong Wang, Junhong Zhang, Yan Gong, Conghua Xie

**Affiliations:** 1 Department of Radiation and Medical Oncology, Zhongnan Hospital of Wuhan University, Wuhan, China.; 2 Department of Radiation and Medical Oncology, Affiliated Sanming First Hospital of Fujian Medical University, Sanming, China.; 3 Department of Radiation Oncology, The Affiliated Hospital of Inner Mongolia Medical University, Hohhot, China.; 4 Department of Pathology, China Three Gorges University Medical College, Yichang, China.; 5 Department of Biological Repositories, Zhongnan Hospital of Wuhan University, Wuhan, China.; 6 Central Laboratory of Xinhua Hospital of Dalian University, Department of Medical Oncology, Xinhua Hospital of Dalian University, Dalian, China.; 7 Tumor Precision Diagnosis and Treatment Technology and Translational Medicine, Hubei Engineering Research Center, Zhongnan Hospital of Wuhan University, Wuhan, China.; 8 Hubei Key Laboratory of Tumor Biological Behaviors, Hubei Cancer Clinical Study Center, Zhongnan Hospital of Wuhan University, Wuhan, China.

**Keywords:** MUC3A, NSCLC, NFκB, Radiosensitivity, DNA damage

## Abstract

Mucin 3A (MUC3A) is highly expressed in non-small cell lung cancer (NSCLC), but its functions and effects on clinical outcomes are not well understood. Tissue microarray of 92 NSCLC samples indicated that high levels of MUC3A were associated with poor prognosis, advanced staging, and low differentiation. MUC3A knockdown significantly suppressed NSCLC cell proliferation and induced G1/S accumulation via downregulating cell cycle checkpoints. MUC3A knockdown also inhibited tumor growth *in vivo* and had synergistic effects with radiation. MUC3A knockdown increased radiation-induced DNA double strain breaks and γ-H2AX phosphorylation in NSCLC cells. MUC3A downregulation inhibited the BRCA-1/RAD51 pathway and nucleus translocation of P53 and XCRR6, suggesting that MUC3A promoted DNA damage repair and attenuated radiation sensitivity. MUC3A knockdown also resulted in less nucleus translocation of RELA and P53 *in vivo*. Immunoprecipitation revealed that MUC3A interacted with RELA and activated the NFκB pathway via promoting RELA phosphorylation and interfering the binding of RELA to IκB. Our studies indicated that MUC3A was a potential oncogene and associated with unfavorable clinical outcomes. NSCLC patients with a high MUC3A level, who should be more frequent follow-up and might benefit less from radiotherapy.

## Introduction

Non-small cell lung cancer (NSCLC) is one of the most common human malignancies with high mortality rate over the past 30 years 1. Approximately 60% of NSCLC patients are diagnosed at advanced stages, and half need radiotherapy during their treatment course. Local control after radiotherapy weights the most to survival benefit 2-5. The ultimate goal of radiotherapy is a high-dose-delivery in the target without radiation-induced injuries in the surrounding normal tissues 6. Previous clinical trials indicate that a radiation dose of >84 Gray (Gy) is required for 50% tumor control within 3 years 7. Although enormous radiotherapeutic techniques boost recently 8, the therapeutic effects of radiotherapy are still unsatisfactory. The 5-years survival rate of NSCLC is only 16.1% 9, 10. Therefore, it is urgent to promote the biological effects of radiation and find a reliable index to predict radiotherapy effects.

Many mucins are reported as biomarkers to identify and monitor the progression of lung cancer. It is aberrantly expressed in NSCLC cells and participates in tumor progression and metastasis via altering various signalling pathways 11-13. Situ et al. reveal that MUC1 is overexpressed in 86.3% adenocarcinomas, 39.1% squamous cell carcinomas, and 74.1% other NSCLC subtypes, and acts as an independent prognostic factor of NSCLC 14, 15. MUC4 is upregulated in patients with lung adenocarcinoma (LUAD) at stages I (138 cases) and II (17 cases), and its high levels in early-stage patients are correlated with an unfavorable prognosis 16. In addition, MUC5AC is elevated in 26.2% (16/61) patients with stage I/II NSCLC and predicts the poor clinicopathological profiles and prognosis 17. These studies suggest a close correlation between mucins and lung cancer pathogenesis, development, and prognosis.

MUC3A is membrane-associated mucin with glycosylation and expressed in various epithelial cells. MUC3A contains a sperm protein, enterokinase, agrin, and epithelial growth factor (EGF) domain and functions through ligand binding and intracellular signalling pathways 18. Favorably, MUC3A is rarely expressed in normal pulmonary epithelial cells, making it been a promising tumor biomarker for lung cancer 19. Moreover, MUC3A exerted oncogenic profiles in breast, pancreatic, gastric, colorectal, prostate, and renal cancers 20-25.

Currently, the mechanism of MUCs' effects on the occurrence and development of NSCLC is still unclear as to the complex biological properties of mucins in a cell type-specific manner 25. In this study, we report that MUC3A exerts oncogenic profiles in NSCLC and may be a promising marker to predict radiotherapeutic effectiveness. The development of a monoclonal antibody targeting MUC3A should be encouraging for fighting against NSCLC.

## Material and Methods

### Tissue microarray and bioinformatics analysis

The lung cancer tissue microarray, containing 92 LUAD tissues and paired para-carcinoma tissues, was purchased from Outdo (Shanghai, China). The samples come from the National Human Genetic Resources Sharing Service Platform (2005DKA21300). All the dots on the chip were detected by immunohistochemistry (IHC) with survival information and included in univariate and multivariate survival analyses. Both the intensity and positive percentages of immunohistochemistry (IHC) were used to examine the MUC3A expression: the IHC H-score (values 0-400) = the scores for intensity of positive staining (less than 5% scored “0”; 5-24% scored “1”; 25-49% scored “2”; 50-74% scored “3”; and more than 74% scored “4”) × the percentage of positive-stained cells × 100. In the cancer tissues of all the 92 cases, the median MUC3A H-score was 140. *Oncomine, Km plotter, and Gepia* were used for bioinformatic analysis.

### Cells

The H1975, A549, H1299, HCC827, H460, and PC9 cells were purchased from the Type Culture Collection (Chinese Academy of Sciences, Shanghai, China) with the short tandem repeat sequencing authentication (Cellcook Biotech, Guangzhou, China, Figure S1-2). Cells were cultured in RPMI-1640 medium (HyClone, USA) with 10% fetal bovine serum (Gibco, Cat#: A4766801, USA), 100 units/ml penicillin and 100 µg/ml streptomycin (HyClone).

### Cell proliferation assay

The cells were seeded in 96-well plates (1,000 cells/well) and cultured for 5 days. After adding 10 μl CCK-8 (Dojindo, Japan) to each well and incubating at 37 °C for 2 h, the absorbance at 450 nm was measured by the Rayto-6000 system (Rayto, China) and normalized to that of RPMI-1640 medium as control.

### Colony formation assay

For cell proliferation, we seeded 50 cells to each well of 12-well plates for 7 days, then fixed with 4% paraformaldehyde (PFA) and stained with crystal violet.

For radiation sensitivity, 100, 200, 400, 1,000, 2,000 and 10,000 cells were seeded in 6-well plates. The cells were then irradiated at 0, 1, 2, 4, 6, 8, and 10 Gy with the Small Animal Radiation Research Platform (SARRP, 204 kV, PXI X-RAD 225Cx, CT, USA). After 15 days, the colonies were fixed with 4% PFA for 15 min and stained with crystal violet. The cells were photographed, and the numbers of colonies were counted. A “multitarget-single hitting” model was applied to fit the survival curve.

### Cell cycle assay

After starving for 6 h, the cells were harvested, then fixed with cold ethanol overnight. After totally removed ethanol, cells were incubated with propidium iodide and RNAse (BD, USA) in the dark for 15 min. The stained cells were assessed by flow cytometry (FACS AriaIII, BD, USA) and analyzed by FlowJo vX.0.7 software.

### Cell apoptosis assay

The cells cultured on 24-mm coverslips were fixed by 4% PFA at room temperature for 30 min. After incubating with 0.1% Triton X-100 for 2 min, the TUNEL assay was performed according to the manufacturer's instruction (Roche, Germany). The nuclei were labeled with DAPI at 2 μg/ml, then analyzed by a fluorescence microscope (Olympus IX 73 DP80, Japan).

### Modified Boyden chamber migration and invasion assay

The cells were seeded into the upper chambers of 12-well plates (1.5 × 10^5^ cells/well) and cultured for 24 h. For invasion assay, the transwell membranes were precoated with Matrigel (1:40 dilution, Corning, USA) at 37 °C. After 24 h, the cells were fixed with 4% PFA and stained with 0.1% crystal violet. The invaded and migrated cells were counted at 5 random fields per chamber under a phase-contrast microscope (DC 300F, Leica, Germany).

### Protein and cytoplasmitc protein extraction

The nuclear and cytoplasmic components were extracted using the NE-PER Nuclear Cytoplasmic Extraction Reagent kit (Pierce, Rockford, IL, USA). Briefly, the treated cells were washed twice with cold PBS, and centrifuged at 1000 rpm for 5 min. The cell pellet was suspended in 200 μl of the cytoplasmic extraction reagent I by vortexing for 5 s. The suspension was incubated on ice for 20 min followed by the addition of 11 μl of the second cytoplasmic extraction reagent II, vortexed for 5 s, incubated on ice for 1 min and centrifuged at 12,000 g for 5 min. The supernatant fraction (cytoplasmic extract) was collected. The insoluble pellet fraction, which contains crude nuclei, was resuspended in 100 μl of nuclear extraction reagent by vortexing for 15 s and incubated on ice for 10 min, then centrifuged at 12,000 g for 10 min. The resulting supernatant, constituting the nuclear extract, was used for the subsequent experiments.

### Immunoblotting

The cells were lysed in RIPA buffer containing protease inhibitor and phosphatase inhibitor (Sigma-Aldrich, USA) on ice for 30 min. The cell lysates were centrifuged at 12,000 g for 15 min, and the supernatants were collected. Co-immunoprecipitation was performed as the protocol of the BeaverBeads™ Protein A/G Kit (Beaverbio, Suzhou, China). The total proteins were separated using 7.5-12.5% SDS-PAGE (Bio-Rad) and transferred to a PVDF membrane (Millipore, USA). TBST with 5% milk was used to block non-specific binding sites. The dilution of antibodies for WB has been list in Table S1-2. The immunoreactive proteins were detected by enhanced chemiluminescence (Thermo Fisher, USA).

### Immunofluorescence

The NSCLC cells were seeded on 24-mm coverslips, fixed with 4% PFA for 30 min, penetrated with 0.1% Triton X-100 and blocked with 5% bovine serum albumin at room temperature for 1 h. After incubated with primary antibodies at 4 °C overnight, the cells were incubated with Cy 3-labelled or FITC-labelled secondary antibodies at room temperature for 1 h. The nuclei were labeled with DAPI (2 μg/ml). The immunofluorescent staining was examined using a fluorescent microscope (IX 73 DP80, Olympus, Japan) or a laser confocal microscope (C2, Nikon, Japan). The mean density was applied to semi-qualified by Image-Pro Plus 6.0.

### Mcherry-GFP-LC3 II autophagy assay

Cells (3 × 10^5^) were seeded in a 6-well plate and transfected with mCherry-GFP-LC3 II (Beyotime Ltd, Beijing, China) adenovirus. The cells were irradiated 4 Gy X-rays 24 h after infection. A fluorescent microscope (Olympus IX 73 DP80, Japan) was used to observe the fluorescence 12 h after irradiation. After the cells were infected with Ad-mCherry-GFP-LC3B adenovirus, mCherry-GFP-LC3B existed in the cytoplasm in the form of diffuse yellow fluorescence (the combined effect of mCherry and GFP), while in the case of autophagy, mCherry-GFP-LC3B gathered on the autophagy membrane in the form of yellow spots (LC3B dot or punctae)). When autophagosomes fuse with lysosomes, they appear in the form of red spots due to the partial quenching of GFP fluorescence.

### Transmission electron microscopy (TEM)

Four hours after irradiation, the cells were washed 3 times with PBS, and collected by a cell scraper. Then, cell suspensions were centrifuged at 2000 r/min for 5 min, and the supernatants were discarded. The cell pellets were fixed with 2.5% glutaraldehyde for 1 h, and then fixed with 1% osmium tetroxide buffer for 1 h. Subsequently, an ascending series of alcohol were performed for dehydration before embedding samples in Araldite. Ultrathin sections were observed with TEM (HT7700, Hitachi, Japan, 100kV).

### Animals

Six-week-old female *BALB-C/null* mice (Vital River Laboratory Animal Technology Co., Ltd, Beijing, China) were housed in a specific pathogen-free, temperature, and humidity-controlled environment. According to the Wuhan University Animal Care Facility and the National Institutes of Health Guidelines, all animal experiments were performed.

### Xenograft tumor model

Approximately 5 × 10^6^ H1975-GFP cells were harvested, resuspended in 100 μl PBS, and injected subcutaneously into each mouse's right flank. Treatment was commenced when the tumor size reached approximately 100 mm^3^. The size of the tumor and the weight of the mice were recorded every day. An animal* in vivo* imaging system was used to evaluate the tumor's size on Days 7 and 14 after radiotherapy. Tumor volume (V) was calculated according to the formula: π/6 × length × width^2^.

The tissues from the tumor-bearing mice were fixed in 4% PFA at 4 °C overnight and embedded into paraffin (Paraplast, Sigma-Aldrich) using a tissue processor (Thermo Fisher Scientific, Loughborough, UK). Paraffin sections (5 µm) were cut with a rotation microtome (Thermo Fisher Scientific, Bremen, Germany). The images were collected by Versa 8 (Leica, Germany). The integrated optical density of IHC sections was calculated by Image-Pro Plus 6.0.

## Results

### The poor survival in lung cancer is associated with the high expression of MUC3A

**Figure 1:**
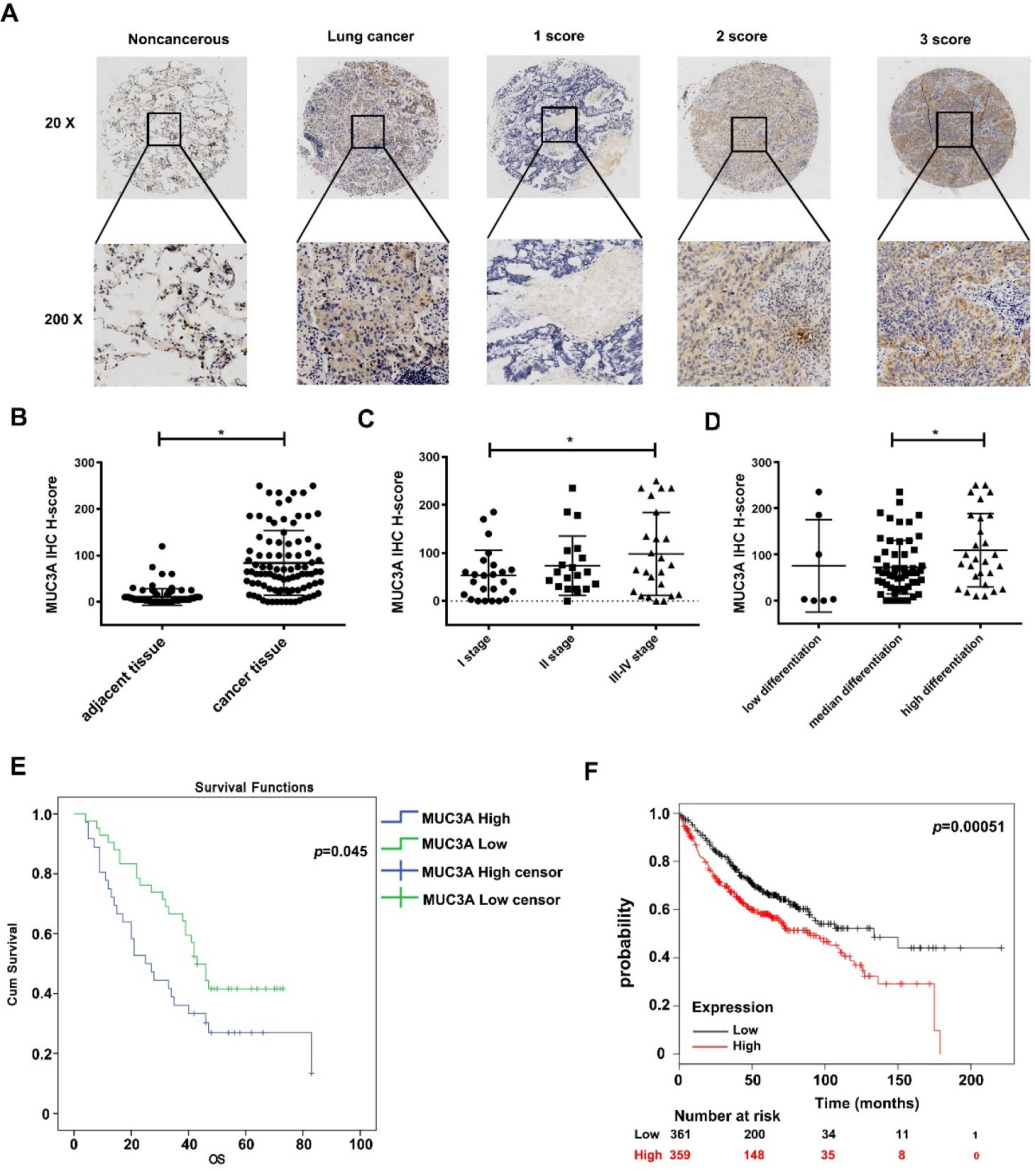
** MUC3A was highly expressed in lung cancer and relevant to the poor survival in database and tissue microarray.** (A) The 20× and 200× representative images of IHC. (B) The expression levels of MUC3A protein were significantly higher in NSCLC tissues than those in the paired normal tissues. The tissue microarray results included 92 pairs (p < 0.001, paired t-test). (C) MUC3A expression levels in lung cancer tissues subgrouped by staging (p < 0.001, ANOVA). (D) MUC3A expression levels in lung cancer tissues subgrouped by differentiation (p < 0.001, ANOVA). (E) Kaplan-Meier plot of 92 patients with survival data (from tissue arrays) stratified by MUC3A expression levels. Patients expressing less MUC3A displayed higher overall survival than the other patients (p = 0.045, Kaplan-Meier survival test). (F) Kaplan-Meier plot of 720 patients with survival data stratified by MUC3A mRNA levels. Patients expressing more MUC3A displayed shorter overall survival than the other patients (p = 0.00051, Kaplan-Meier survival test).

From the public database, we found that MUC3A levels in LUAD and squamous cell carcinoma were significantly higher than those in normal lung tissues (p < 0.01, Figure S3A). We performed a LUAD tissue microarray (Figure S3B) with a particular scoring system (Figure 1A). Demographic characteristics and pathological baseline of tissue-chip were listed in Table 1, showing that the MUC3A was significantly elevated in LUAD tissues (p < 0.001, Figure 1B). Samples were split into 2 groups by the best cut-off value at H-score 140, and MUC3A was positively associated with advanced pathological stages (Figure 1C) and differentiation (Figure 1D). Moreover, high MUC3A expression levels predicted shorter survival in both tissue-chip (median survival 47.5 versus 54.5 months, p = 0.045, Figure 1E) and public database (http://kmplot.com, p = 0.00051, Figure 1F). Surprisingly, tumor size and lymphatic metastasis status failed to correlate with MUC3A expression levels (Figure S3C, D).

### Statistical analyses

Each experiment was performed in triplicates, and data presented in the representation of 3 individual experiments. A two-tailed Student's t-test and one-way analysis of variance (ANOVA) were used to evaluate different groups' statistical significance. Statistical analyses were performed with SPSS 16.0. *P* < 0.05 were considered as statistical significance.

We further verified the expression of MUC3A in 6 human NSCLC cell lines, including H1975, A549, H1299, HCC827, H460, and PC9. Among these cell lines, LUAD, H1975 and H1299 cells we observed to be expressing high amount of MUC3A and therefore, we have used these two cells line for our further exploration.

### MUC3A knockdown attenuated NSCLC cell proliferation, migration, and invasion

**Figure 2:**
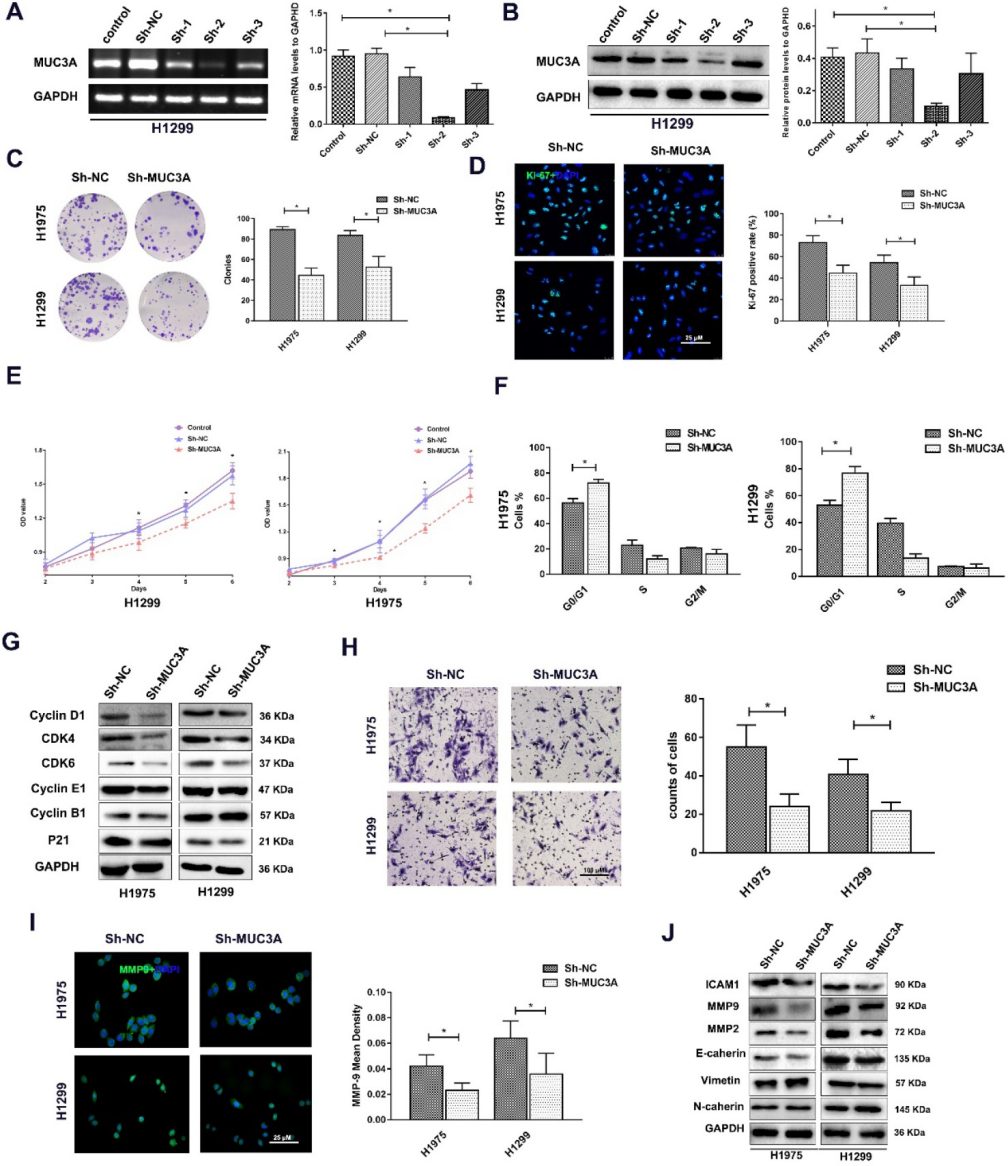
** MUC3A knockdown attenuated NSCLC cell proliferation, migration, and invasion.** (A) PCR analysis to detect the knockdown effects of different shRNAs. shRNA-2 was more efficient than the others in H1299 cells. (B) WB analysis to detect the knockdown effects of different shRNAs in H1299 cells. (C) Representative images of crystal violet stain on Day 15. (D) Representative images of Ki-67 IF staining in H1975 and H1299 cells. (E) CCK-8 array to detect cell proliferation. (F) FACS analysis to detect the cell cycle distribution. More cells in the MUC3A knockdown group were accumulated at G0/G1. (G) Immunoblotting analysis of protein abundance of Cyclin D1, CDK4, CDK6, Cyclin E1, Cyclin B1, p21. (H) Transwell assay for cell migration and invasion. The MUC3A knockdown group had significantly fewer migration and invasion cells than the control group. (I) Representative images of MMP9 IF staining in H1975 and H1299 cells. MUC3A knockdown reduced MMP9 expression. (J) Western blotting analysis of protein abundance of ICAM1, MMP2, MMP9, E-cadherin, Vimentin, and N-cadherin. *, p < 0.05.

Lentiviruses carrying MUC3A shRNAs were used to obtain MUC3A-knockdown H1975 and H1299 cells (Figure 2A, B). The ability of colony formation was notably impaired after knockdown of MUC3A gene (Figure 2C). Interestingly, the Ki-67 index and CCK-8 assay indicated that MUC3A knockdown remarkably attenuated the cell proliferation (Figure 2D, E). We further investigated the cell cycle attribution and found that MUC3A knockdown caused the cells arrested at the G1/S phase with the depletion of Cyclin D1, CDK4, and CDK6 (Figure 2F, G).

In addition, impaired function of MUC3A abrogated cell migration (Figure 2H) with decreased expression of MMP-2 and MMP-9 (Figure 2I, J). However, the size and shape of NSCLC cells, as well as proteins involved in epithelial-to-mesenchymal transition were not changed after MUC3A knockdown (Figure 2I, J).

### MUC3A knockdown inhibited the activity of the NFκB pathway

**Figure 3:**
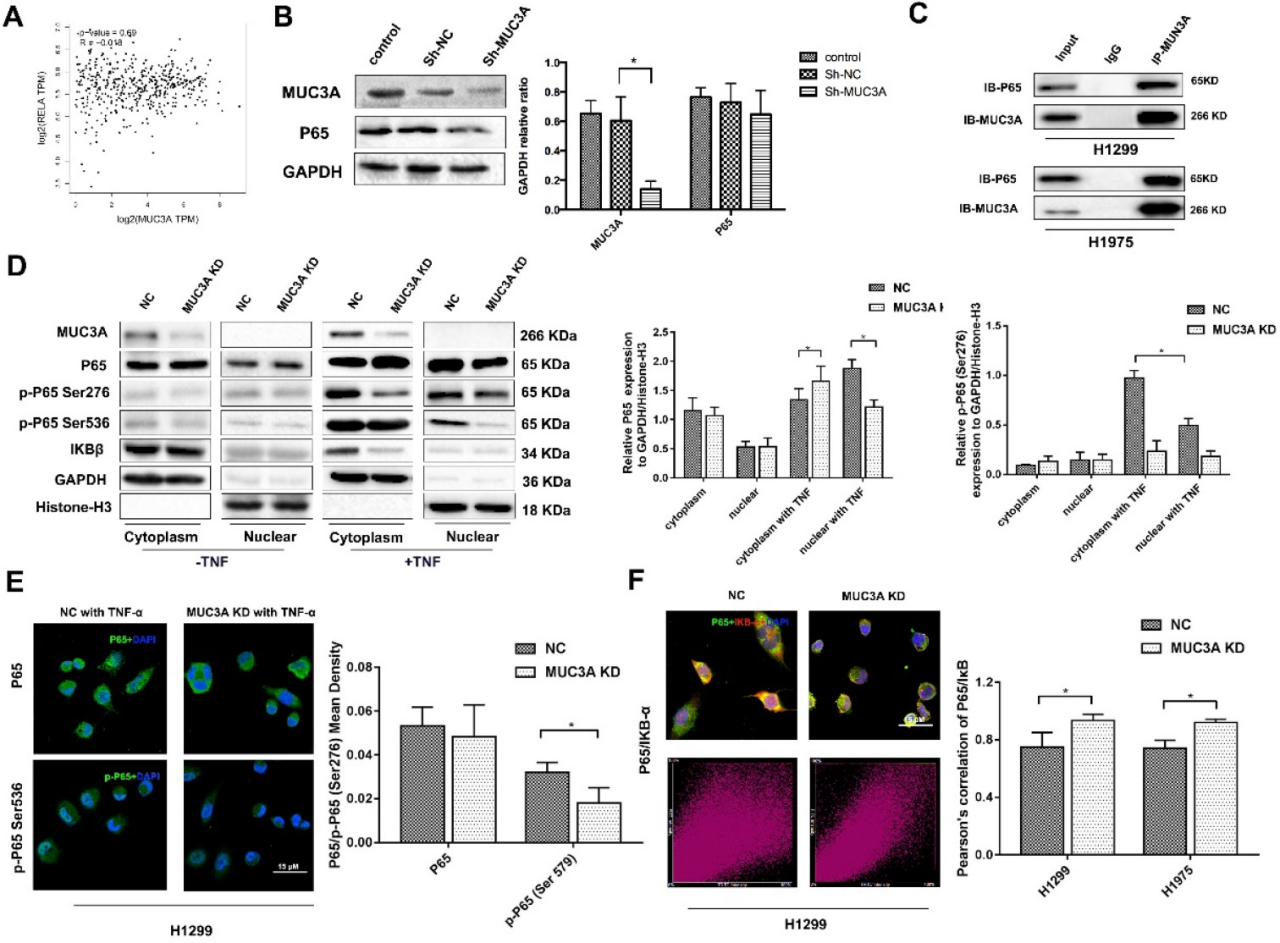
** MUC3A knockdown reduced the activity of the NF-κB pathway.** (A) Pearson correlation between MUC3A and P65 log2 expression. p = 0.69, R = -0.018. (B) WB to detect the relationship between MUC3A and p65. (C) Co-IP to analyze the binding of MUC3A and p65. The representative images exhibited that MUC3A was linked to p65. (D) WB was used to investigate the MUC3A, p65, p-p65, IκB, GAPDH, and Histone H3. (E) Representative images of p65 and p-p65 IF staining in H1299 cells. (F) IF to detect the binding condition of p65 (green) and IκB (red). The scatter image presented the correlation of fluorescence intensity between FITC and TRITC. *, p < 0.05.

We utilized the GEPIA online tool and found that there was no correlation between the MUC3A and P65 (Figure 3A). We confirmed that MUC3A knockdown could not affect the P65 expression in whole-cell lysis (Figure 3B). However, the protein-protein interaction database indicated that P65 could interfere with MUC3A based on a high-throughput affinity chromatography analysis. We further confirmed the physical interaction between P65 and MUC3A in H1299 and H1975 cells via co-immunoprecipitation (Figure 3C).

Continuously, we found there was only a slightly higher ratio of cytoplasm and nucleus P65 expression in MUC3A knockdown cells. However, once the cells were treated with a P65 activator, tumor necrosis factor α (TNF-α), at 10 ng/ml for 30 min, P65 was rapidly phosphorylated and attenuated in both nuclei and cytoplasm of MUC3A-knockdown cells. Furthermore, a very low amount of IκB was accumulated in MUA3A-knockdown cells even after TNF-α stimulation (Figure 3D). Favorably, we obtained the same findings via immunofluorescence and semi-qualified by laser confocal microscopy (Figure 3E). The IκB and P65 laser scatter plots were linearly formed in MUC3A knockdown cells (Figure 3F), indicating a colocalization for IκB and P65. Moreover, there was a weak binding observed between IκB and P65 in MUC3A-knockdown cells, suggesting that the NFκB pathway was attenuated by MUC3A (Figure S4A-C).

### MUC3A knockdown contributed to a severe DNA damages induction upon the exposure of X-rays

**Figure 4:**
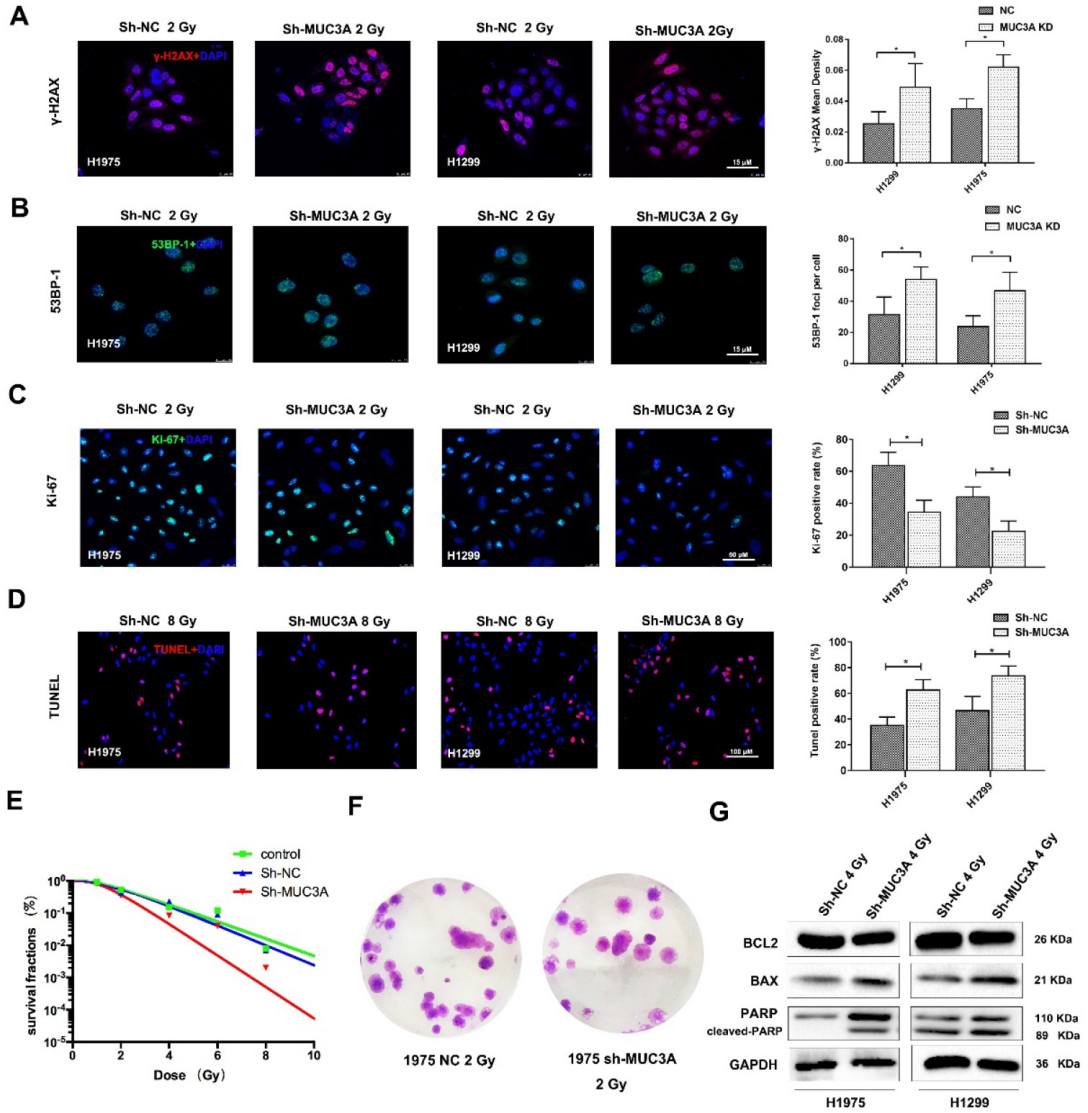
** MUC3A knockdown induced more DNA damages by X-rays.** (A) Representative images of γ-H2AX IF staining in H1975 and H1299 cells. (B) Representative images of 53BP1 IF staining in H1975 and H1299 cells. (C) Representative images of Ki67 IF staining in H1975 and H1299 cells. (D) Representative images of TUNEL staining in H1975 and H1299 cells. (E) The multitarget-single-hitting model was used to fit the survival curve. The survival fraction of MUC3A knockdown cells was significantly lower than that of the control groups. (F) Representative crystal violet staining photos of H1975 parental and MUC3A knockdown cells with 2 Gy irradiation. (G) Western blotting analysis of BLC2, BAX, and PARP protein levels. *, p < 0.05.

Previously, siRNA screening reported that MUC3A silencing would elevate the cellular level of γ-H2AX. 26. Both MUC3A-wild type and knockdown cells were irradiated with 2 Gy X-rays and then fixed for 30 min. As a result, we observed that MUC3A knockdown cells induced more DNA damages and promoted radiation effects more significant than those of the wild type cells (Figure 4A-C). We further verified our results by TUNEL assay, however, here we have used 10 Gy X-rays to irradiate the cells to cope up with sensitivity limitations of the TUNEL assay. The TUNEL results confirmed the synergistic effects of MUC3A knockdown and irradiation on DNA damage (Figure 4D). The survival curve indicated that MUC3A-knockdown cells were more radiosensitive than those of the wild type (Figure 4E-F). Moreover, immunoblotting results showed that MUC3A-knockdown cells exposed to 2 Gy X-rays had increased BAX expression levels and cleaved-PARP, indicating an elevation in cell death (Figure 4G).

**Figure 5:**
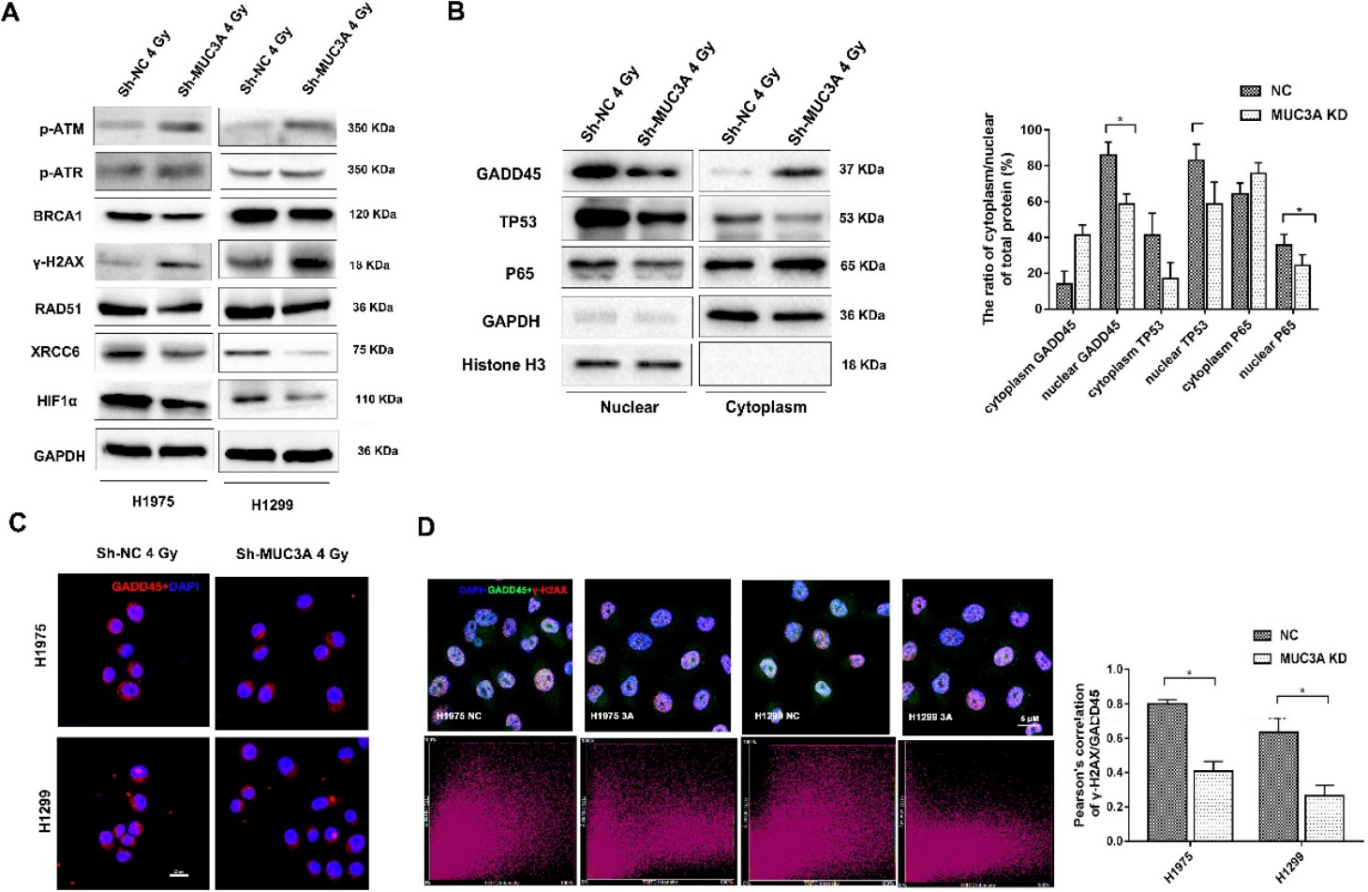
** The mechanism of MUC3A knockdown induced more DNA damages by X-rays.** (A) Immunoblotting analysis of p-ATM, p-ATR, γ-H2AX, BRCA1, RAD51, XCRR6, and HIF1α protein levels after 4 Gy irradiation. (B) Immunoblotting analysis to investigate the plasm and nucleus protein levels of GADD45, TP53, and p65. (C) Representative images of GADD45 in H1975 and H1299 cells after 4 Gy irradiation. (D) As previously described, specific software was used to analyze the co-localization of γ-H2AX and DADD45. In MUC3A-knockdown cells, less GADD45 was recruited to γ-H2AX. *, p < 0.05.

Higher expression levels of γ-H2AX, p-ATM, and p-ATR were observed in MUC3A-knockdown cells after 2 Gy irradiation (Figure 5A). In addition, the BRCA1 and RAD51 repair axis was blocked in MUC3A-knockdown cell along with the suppression of XRCC6 in MUC3A-knockdown cells after irradiation. Also, HIF-1α, p-P65, and p-P53 were decreased in MUC3A-knockdown cells after 2 Gy irradiation suggesting a severe DNA damage in MUC3A-knockdown cell.

Additionally, we investigated the transcriptional regulators and their localization including S5A, P65, P53, and GADD45 and found that these proteins were decreased in the nucleus after losing the function of MUC3A (Figure 5B) suggesting that the MUC3A has a key role in translocation of these marker from cytosol to nucleus. On the other hand, RAD51 and XRCC6 were observed to be located in the nucleus in wild type cells and were absent in the nucleus of MUC3A-knockdown cells (Figure S5B, C). Interestingly, MUC3A knockdown impaired GADD45 nucleus translocation (Figure 5C) with less binding with γ-H2AX, indicating less DNA damage repair activities initiated in the MUC3A-knockdown cells after irradiation (Figure 5D).

Additionally, the exposure of 4 Gy irradiation to the MUC3A knockdown cells induced the late stage of autophagy as evident by the accumulation of red dots (Figure S5D). Similarly, immunoblotting results also showed higher LC3 II and lower P62 protein levels (Figure S5E) along with the accumulation of significantly higher autophagic vacuole in MUC3A-knockdown cells after irradiation (Figure S5F). Unfortunately, our results were unable to support that MUC3A facilitated radio sensitivity via affecting cellular autophagy as Beclin-1 and ATG-5 did not accumulate in MUC3A-knockdown cells.

### MUC3A knockdown impaired tumor growth and promoted radiosensitivity *in vivo*

**Figure 6:**
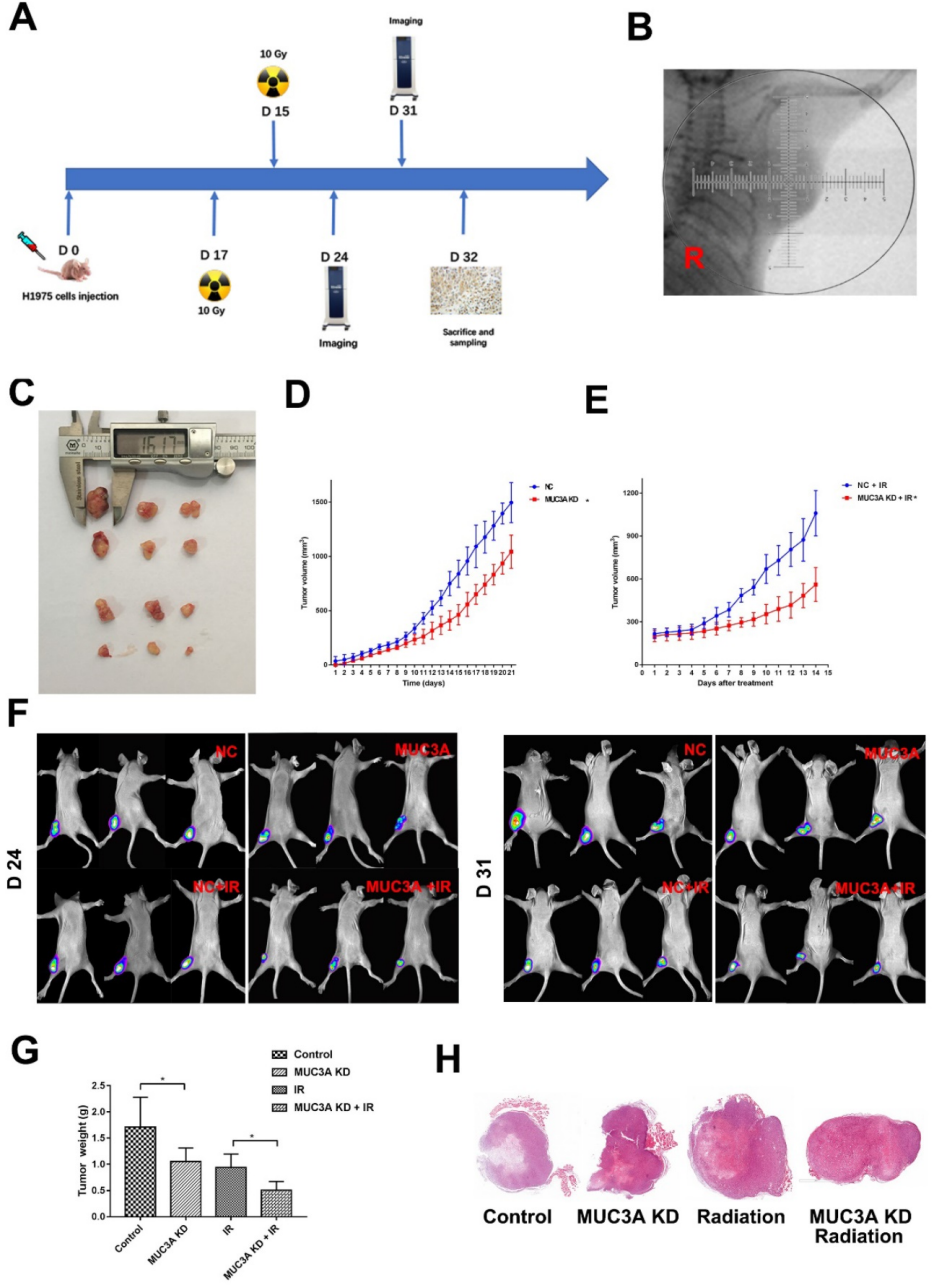
** MUC3A knockdown impaired tumor growth and promoted radiosensitivity* in vivo.*** (A) Treatment schema. (B) Gross view of the tumors. (C) Growth curve of tumor volume for the group without radiotherapy. (D) Growth curve of tumor volume for the group with radiotherapy. (E) *In vivo* imaging of the size and destiny of GFP-H1975 cells on Days 24 and 31. (F) Tumor weight was significantly different between the control and MUC3A knockdown groups with or without radiotherapy. (G) HE staining was applied to investigate the size and shape of tumor and pathological characters. All the masses were identified as a malignant tumor, and the shapes were similar to H1795 cells. More necrosis was observed in the control group. *, p < 0.05.

*BALB-c/Null* mice were subcutaneously implanted with H1975-NC-GFP or H1975-MUC3A KD-GFP cells in the right flank (Figure 6A). Approximately 2 weeks after implantation, the tumor size reached 150 mm^3^, and at this time point the mice received radiation or mock treatment. MUC3A knockdown significantly suppressed tumor volume. The combination of radiation exerted synergistic effects on tumor reduction at both Days 7 and 14 post-treatment (Figure 6B-F). All the masses were identified as malignant tumors via HE staining (Figure 6G).

**Figure 7:**
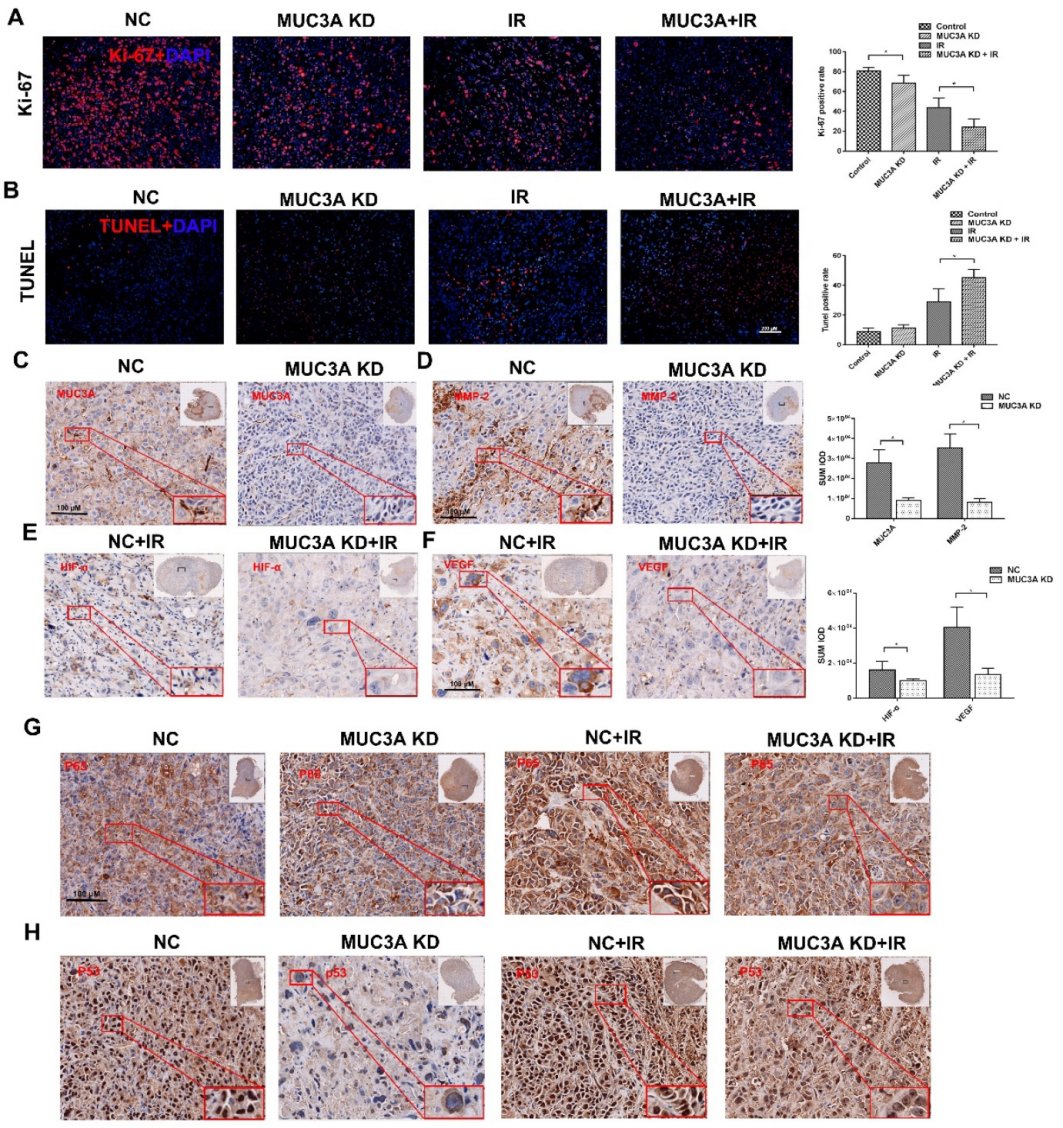
** Knockdown of MUC3A presented a comprehensive anti-tumor effect *in vivo*.** (A) Representative images of Ki-67 IF staining in tumor tissues. (B) Representative images of TUNEL staining in tumor tissues. (C) Representative IHC images of MUC3A. The MUC3A knockdown cells had less MUC3A expression than the control. (D) Representative IHC images of MMP2. (E) Representative IHC images of HIF1α. (F) Representative IHC images of VEGF. (G) Representative IHC images of P65. (H) Representative IHC images of TP53. *, p < 0.05.

Favorably, MUC3A knockdown and X-rays exerted significantly synergetic effects *in vivo* (Figure 7A, B). The verification of knock down of MUC3A gene via shRNA lentivirus infection was done by IHC (Figure 7C). Similar to the findings *in vitro,* MMP-2 was notably decreased in the MUC3A-knockdown tumor (Figure 7D). HIF-1α and VEGF expression levels were attenuated in MUC3A-knockdown tumors after irradiation (Figure 7E-F).

MUC3A knockdown upon exposure of X-ray impaired the unclear translocation of P65 and decreased P65 expression (Figure 7G). MUC3A-wild type cells expressed more P53 in the cytoplasm and nucleus, while P53 was rarely expressed in the nucleus of MUC3A-knockdown cells (Figure 7H). On the other hand, the groups with X-ray treatment, exhibited the significant up regulation of P53 in both MUC3A-wild type and MUC3A-knockdown cells. Furthermore, the nucleus location of P53 was less in MUC3A-knockdown cells.

## Discussion

MUC3A is a membrane-associated MUC with abnormal expression in various cancers and may involve in tumorigenesis and progression 27. Our results and public database also bolster the capacity for MUC3A to predict the clinical outcomes of NSCLC. The mechanism of MUC3A upregulation in cancers remains unclear. It was reported that a conserved TFLK motif and hypoxia tumor microenvironment were attributed to MUC3A expression. MUC3A share the same sperm protein, enterokinase, and agrin domain with MUC1, suggesting that its autoproteolysis may affect cell migration and PI3K-Akt pathway activation by EGF family phosphorylation 20.

In the MUC3A-knockdown model, we observed that cell growth was inhibited *in vitro* and *in vivo*. Herein, we demonstrated that MUC3A knockdown interfere with the cell cycle process as well. Previous studies have reported that cyclinD1 is one of the downstream gene of NFκB regulating the G1/S transit via downregulating the CDK4/6, which induces cell accumulated in G1/S phase 28, 29. FACS results presented S phase were manifestly reduced when MUC3A knockdown along with decreased expression of CyclinD1, CDK4/6. Our finding suggests that MUC3A promotes cell proliferation by aberrant regulation of the G1/S checkpoint.

RELA, also called P65, a critical member of NFκB unit, was observed to interfere with MUC3A in the present study. NFκB is a regulator to control cell growth and survival and constitutively active in various human malignant tumors 28, 30-33. P65 nucleus translocation and phosphorylation are two crucial behaviors of the canonical activation process of the NFκB pathway 34-36. In MUC3A-knockdown model, under TNF stimulation, the NFκB-p65 nucleus translocation was dramatically impaired.

Meanwhile, both p-NFκB-p65 (Ser276) and p-NFκB-p65 (Ser536) were dephosphorylated in the cytoplasm and nucleus. Furthermore, we investigated more IκB protein bound to P65 and MUC3A may contribute to maintaining the phosphorylation of P65. Therefore, p-NFκB-p65 (Ser276) was dephosphorylated in MUC3A knockdown cells and enhanced the stability of the P65/IκBβ complex. These findings indicate that the interaction of MUC3A and P65 is essential to phosphorylate and nucleus translocation of P65, thus mediates NFκB activation in NSCLC cells.

High energy X-rays inflict cellular damages directly via ionizing water molecules and producing hydroxyl radicals to attack the DNA 37. The X-rays can induce various forms of DNA damage, and DSBs are determinants of cellular radiosensitivity. However, 53BP1, GADD45, and γH2AX are the indexes that exhibit DNA linkage and reflect DNA damage 38-41. Additionally, homologous recombinational repair (HRR) and non-homologous end-joining (NHEJ) are two major pathways to repair DSBs. In mammalian cells, HRR prefers to DSBs, BRCA/Rad51 axis is active by p-ATM and plays a vital role in repairing DNA damage 42, 43; our findings indicated a noticeable interruption of the BRCA/Rad51 axis. In addition, XRCCs are involved in NHEJ, and MUC3A knockdown also attenuates XRCCs in NSCLC cells 44. In this study, MUC3A knockdown remarkably downregulated BRCA1 and RAD51 translocation, suggesting that the radiosensitivity was promoted by suppression of DNA damage repair and activation of pro-apoptotic proteins. Together, these findings revealed a potential mechanism for radiosensitive enhancement.

As we know, the activation of p53 can occur in response to DNA damage. Ionizing radiation induced DSB triggers p53 activated with a rapid accumulated around DNA foci and mediate transcriptional activation which promotes cell-cycle arrest, apoptosis, or DNA repair 45-48. In our study, we observed that MUC3A knockdown impair P53 nuclear translocation. P53 is binding to 53BP1 rapidly when DSS generated, then provide a repair platform for DDR. As previously described, the function of BRCA/Rad51 axis and NHEJ were significantly interrupted, which was attribute to loss-of-function P53.

Furthermore, we noted that MUC3A would induce angiogenesis in NSCLC via elevating the expression of VEGF. In this study, we assume VEGF was regulated in two ways: NFκB signaling induces angiogenesis by increasing the production of VEGF 49-51, and radiation-mediated hypoxia triggers HIF-1α transcription and upregulates VEGF expression 51, 52. MUC3A knockdown may enhance radiosensitivity by promoting oxygen stress and impair HIF-1α and VEGF expression levels.

MMP-2 and MMP-9 are also known to stimulate tumor angiogenesis and EMT through partial proteolysis of the ECM 53-57. Our results failed to illustrate NSCLC cells' EMT; however, cell-cell attachment showed closer in MUC3A knockdown cells. The alterations suggest that MUC3A promotes the migration and invasion of NSCLC cells in various ways. We found that high MUC3A expression was a trend to indicate more lymph node metastasis.

In conclusion, our studies indicated that MUC3A promoted tumorigenesis via activating the NFκB pathway and impaired X-rays response via interrupting DNA damage repair. Its high expression was associated with unfavorable clinical outcomes in patients with lung adenocarcinoma, who should be more frequent follow-up and benefit less from radiotherapy. While targeting the MUC3A protein inhibition or deletion may contribute to enhance survival and suppression of lung tumorigenesis.

## Supplementary Material

Supplementary figures and table.

## Figures and Tables

**Table 1:** MUC3A expression and demographic and clinicopathological characteristics

	MUC3a		N	p-value
	low	high		
**Gender**				
Male	38	13	51	0.300
Female	35	6	41	
	73	19		
**Age**				
≤ 60	37	1	38	0.000
> 60	36	18	54	
**Tumor size (cm)**				
< 4	33	12	45	0.623
≥ 4	23	11	34	
None	7	6		
**Histological grade**				
I/I-II	5	2	7	0.039
II	43	7	50	
II-III/III	22	9	31	
I-III	3	1	4	
**Clinical Stage**				
I	20	3	23	0.045
II	15	3	18	
III-IV	18	8	26	
Non	18	7	25	
**Lymph node status**				
< 4	23	2	25	0.149
≥ 4	48	12	60	
Non	2	5	7	
**Carcinoma**				
Primary	73	19	92	
Adjacent	89	0	89	
**ALK**	67	17	84	0.111
positive	61	13	74	
negative	6	4	10	
Non	6	2	8	
**EGFR**	73	19	92	0.553
positive	17	4	21	
negative	56	15	71	
