# MUC3A promotes non-small cell lung cancer progression via activating the NFκB pathway and attenuates radiosensitivity

**DOI:** 10.7150/ijbs.79140

**Published:** 2022-11-03

**Authors:** Yingming Sun, Xiaoge Sun, Chengcheng You, Shijing Ma, Yuan Luo, Shan Peng, Fang Tang, Xiaoli Tian, Feng Wang, Zhengrong Huang, Hongnv Yu, Yu Xiao, Xiaoyong Wang, Junhong Zhang, Yan Gong, Conghua Xie

**Affiliations:** 1 Department of Radiation and Medical Oncology, Zhongnan Hospital of Wuhan University, Wuhan, China.; 2 Department of Radiation and Medical Oncology, Affiliated Sanming First Hospital of Fujian Medical University, Sanming, China.; 3 Department of Radiation Oncology, The Affiliated Hospital of Inner Mongolia Medical University, Hohhot, China.; 4 Department of Pathology, China Three Gorges University Medical College, Yichang, China.; 5 Department of Biological Repositories, Zhongnan Hospital of Wuhan University, Wuhan, China.; 6 Central Laboratory of Xinhua Hospital of Dalian University, Department of Medical Oncology, Xinhua Hospital of Dalian University, Dalian, China.; 7 Tumor Precision Diagnosis and Treatment Technology and Translational Medicine, Hubei Engineering Research Center, Zhongnan Hospital of Wuhan University, Wuhan, China.; 8 Hubei Key Laboratory of Tumor Biological Behaviors, Hubei Cancer Clinical Study Center, Zhongnan Hospital of Wuhan University, Wuhan, China.

In the version of this article initially published, there was an error in Figure 5D. When organizing the figure with Adobe Illustrator, we used the same picture to make sure the position is the same. However, due to our carelessness, we forgot to adjust the order of the layers, leading to the covered old one rather than the new picture, as in the published figure 5 D, the 2^nd^ and 4^th^ images in the upper panel were the same. We now replace the old 4^th^ image (red arrow) in new figure 5 D. This replacement does not change the conclusion of this study. Below is the figure for erratum.

## Figures and Tables

**Figure 5:**
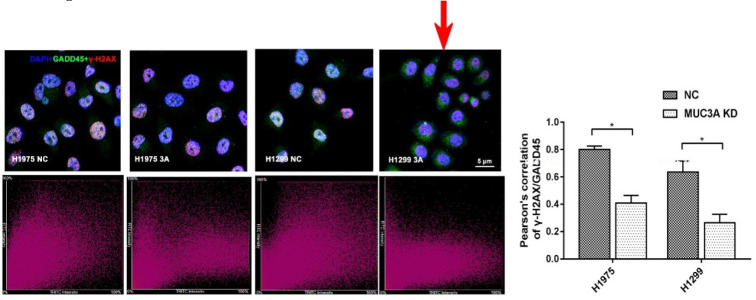
**D**. Corrected image.

