# Supplementary material for: MUC3A promotes non-small cell lung cancer progression via activating the NFκB pathway and attenuates radiosensitivity
Source: Int J Biol Sci. 2021 Jun 16;17(10):2523–36. doi: 10.7150/ijbs.59430 (PMC8315024; doi:10.7150/ijbs.59430)
Supplement: Supplementary file 1 — Supplementary figures and table. [file ijbsv17p2523s1.pdf]

Supplementary materials

Table S1. List of primary antibodies.

**Antigen**	**Species**	**Dilution (IF)**	**Dilution (WB)**	**Supplier**
53BP1, human	Rabbit, polyclonal	1:200	-	Abcam, Cat. #ab36823
Ki-67, human/mouse	Rabbit, monoclonal	1:200	-	Abcam, Cat. #ab16667
P65, human/mouse	Mouse, monoclonal	1:100(IHC)	1:1000	Proteintech, #66535-1-Ig
p-P65(Ser279), human/mouse	Rabbit, monoclonal		1:500	Abclonal, # AP0123
p-P65(Ser536), human/mouse	Rabbit, monoclonal	1:100	1:1000	CST, Cat. #3033T
IκB	Rabbit, monoclonal	1:100	1:1000	Proteintech, Cat. #10268-1-AP
γ-H2AX, human	Mouse, monoclonal	1:100	1:2000	CST, Cat. #80321
p-ATM, human	Rabbit, monoclonal	-	1:500	Abcam, Cat. #ab81292
BRCA1, human	Mouse, monoclonal	-	1:1000	Abcam, Cat. #ab16781
RAD51, human	Rabbit, polyclonal	1:100	1:1500	Proteintech, Cat. #14961-1-AP
XCRR5, human	Rabbit, polyclonal	-	1:1000	Proteintech, Cat. #14961-1-AP
XCRR6, human	Rabbit, polyclonal	-	1:1000	Proteintech, Cat. #16389-1-AP
GADD45, human	Rabbit, polyclonal	1:100	1:1000	Proteintech, Cat. # 13747-1-AP
P53, human/mouse	Rabbit, polyclonal	1:150(IHC)	1:2000	Proteintech, Cat. # 10442-1-AP
pP53, human	Rabbit, polyclonal	-	1:1000	Abcam, Cat. #ab1431
LC3, human	Rabbit, polyclonal	-	1:1000	Proteintech, Cat. #14600-1-AP
ATG5, human	Rabbit, polyclonal	-	1:1000	Proteintech, Cat. #10181-2-AP
Beclin1, human	Rabbit, polyclonal	-	1:1000	Proteintech, Cat. # 11306-1-AP
GAPDH, human	Rabbit, polyclonal	-	1:10000	Proteintech, Cat. #10494-1-AP
BCL2, human	Rabbit, monoclonal	-	1:2000	Abcam, Cat. #32124
BAX, human	Rabbit, monoclonal	-	1:1000	Proteintech, Cat. #50599-2-Ig
PARP, human	Rabbit, monoclonal	-	1:2000	CST, Cat. #9532
Caspase-3, human	Rabbit, polyclonal	-	1:1500	Proteintech, Cat. #19677-1-AP
Caspase-8, human	Rabbit, polyclonal	-	1:1500	Proteintech, Cat. #13423-1-AP
MMP2, human/mouse	Rabbit, polyclonal	1:100 (IHC)	-	Proteintech, Cat. #10373-2-AP
MMP9, human	Rabbit, monoclonal	-	1:1000	Proteintech, Cat. #10375-2-AP
ICAM1, human	Rabbit, monoclonal	1:100	-	Proteintech, Cat. #10831-1-AP
E-cadherin, human	Mouse, monoclonal	-	1:1000	CST, Cat. #3195
N-cadherin, human	Rabbit, monoclonal	-	1:1000	CST, Cat. #13116
Vimentin, human	Rabbit, monoclonal	-	1:1000	CST, Cat. #5741
VEGF, mouse	Rabbit, monoclonal	1:100 (IHC)	-	Abcam, Cat. #ab32152

Table S2. List of secondary antibodies and counterstaining of nuclei.

The secondary detection system used	Host	Method	Dilution	Supplier
Anti-Mouse-IgG (H + L)-HRP	Goat	WB	1:10000	Proteintech, Cat. #SA00001-1
Anti-Rabbit-IgG (H + L)-HRP	Goat	WB	1:10000	Proteintech, Cat. #SA00001-2
Anti-Rabbit-IgG (H + L)-Alexa Fluor 488	Goat	IF	1:500	Proteintech, Cat. #SA00006-3
Hoechst 33342 nucleic acid staining (DAPI)	-	IF	2 μg/ml	Sigma, Cat. #D8417

Guangzhou Cellcook Biotech Co., Ltd

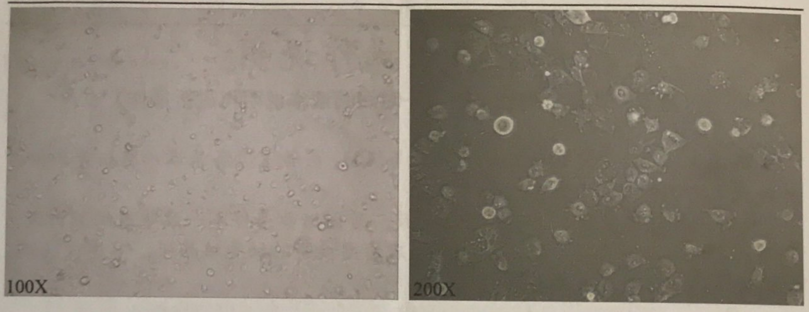

Catalog No.: CC0203

Cell Name: NCI-H1299

Size: T25 culture flask, 1×10^{6}cells

Morphology: Epithelial

Culture Properties: Adherent

Characteristics: The cell line was established from a lymph node metastasis of the lung from a patient who had received prior radiation therapy. The cells have a homozygous partial deletion of the p53 protein, and lack expression of p53 protein. They reported to be able to synthesize the peptide neuromedin B (NMB) at 0.1 pmol/mg protein, but not the gastrin releasing peptide (GRP).

Culture Method: RPMI-1640 10%FBS

Subcultivation Ratio: 1:3~1:6; Twice per week

Trypsined Time: 3-5 minutes

STR Profile:

STR Profile	AMEL	CSF1PO	D13S317	D16S539	D5S818	D7S820	TH01	TPOX	vWA
NCI-H1299	X	12	12	12 13	11	10	6 9.3	8	16 17 18

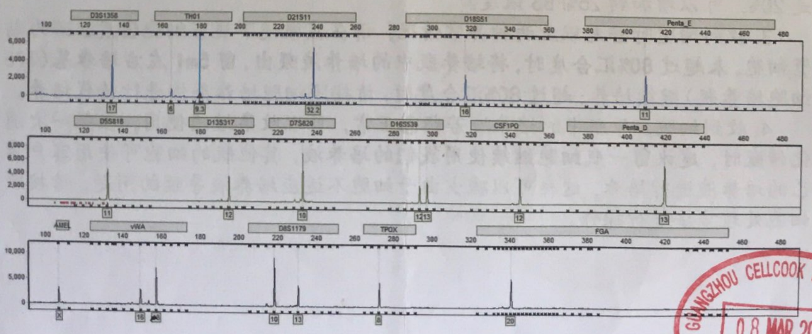

GUANGZHOU CELLCOOK BIOTECH CO.,LTD 08 MAR 2017 CELL STR PROFILING PASS

Address: R&D Unit 602, Stadard Property Unit 3, International Biotech Island, Guangzhou
Web: www.cellcook.com Tel: +8620-84298069 E-mail: info@cellcook.com

Figure S1. Authentication of the H1299 cell line. PCR was amplified with STR Multi-amplification KIT (PowerPlexTM 16HS System). No loci have tri-alleles or tetra-alleles. The contamination of other human cells was not found. The cell STR profiling passed on 2017/3/8.

Cellcook 赛库生物 Guangzhou Cellcook Biotech Co., Ltd

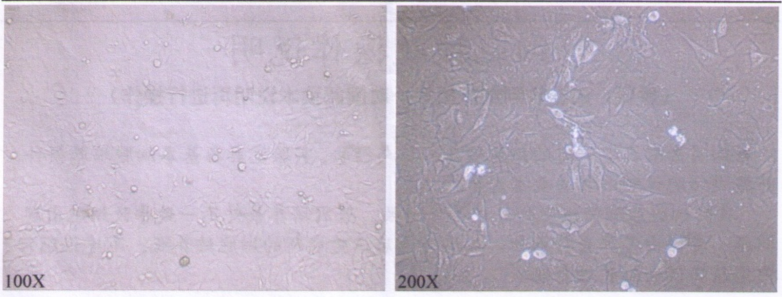

Catalog No.: CC0206

Cell Name: NCI-H1975

Size: T25 culture flask, 1×10^6cells

Morphology: Epithelial

Culture Properties: Adherent

Characteristics: The line was established in July 1988. The tissue donor was a non-smoker.

Culture Method: RPMI-1640 10%FBS

Subcultivation Ratio: 1:3~1:6; Twice per week

Trypsined Time: 3-5 minutes

STR Profile:

STR Profile	AMEL	CSF1PO	D13S317	D16S539	D5S818	D7S820	TH01	TPOX	vWA
NCI-H1975	X	12	10, 13	9, 12	11, 12	8, 11	7	8, 11	18

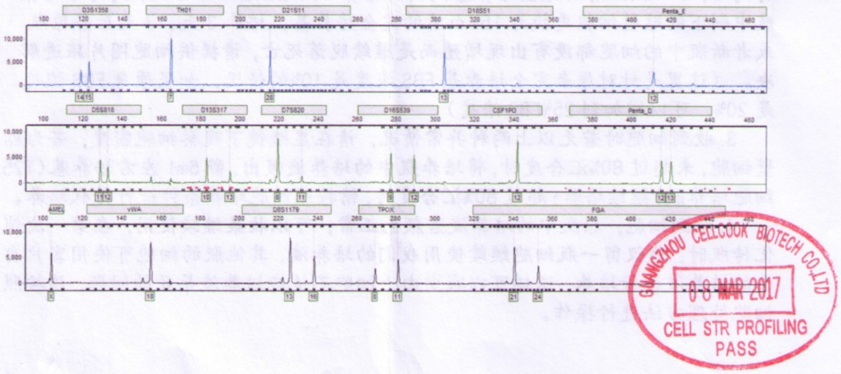

GUANGZHOU CELLCOOK BIOTECH CO.,LTD 08 MAR 2017 CELL STR PROFILING PASS

Address: R&D Unit 602, Stadard Property Unit 3, International Biotech Island, Guangzhou
Web: www.cellcook.com Tel: +8620-84298069 E-mail: info@cellcook.com

Figure S2. Authentication of the H1975 cell line. PCR was amplified with STR Multi-amplification KIT (PowerPlexTM 16HS System). No loci have tri-alleles or tetra-alleles. The contamination of other human cells was not found. The cell STR profiling passed on 2017/3/8.

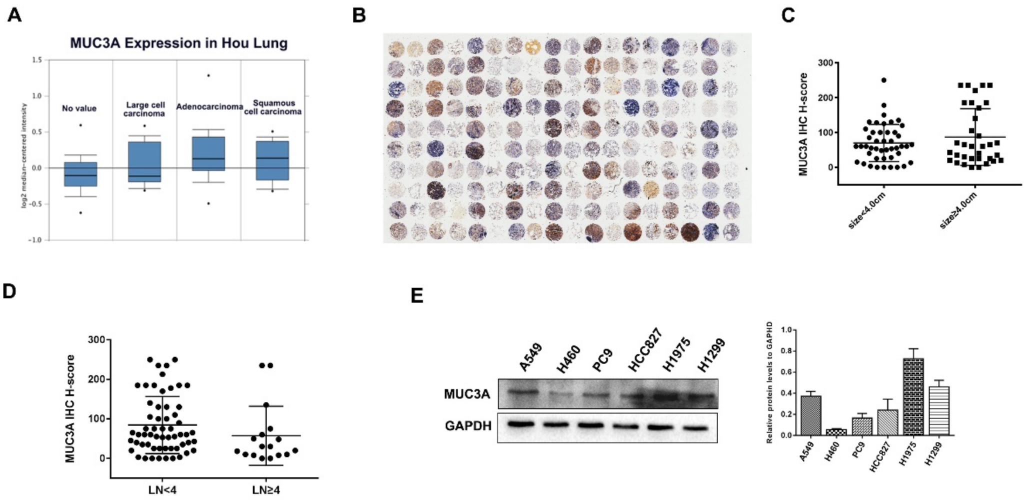

Figure S3. Microarray and MUC3A expression in various NSCLC cell lines. (A) MUC3A expression in the online database of Hou Lung. The levels of MUC3A was higher in adenocarcinoma than others. (B) The HE overview of tissue microarray to primarily verify the adjacent and cancer tissues. (C) MUC3A expression in lung cancer tissues subgrouped by size ($p > 0.05$). (D) MUC3A expression in lung cancer tissues subgrouped by lymph node metastasis ($p > 0.05$). (E) Representative WB images of MUC3A in different NSLCL cell lines. MUC3A was highly expressed in H1975 and H1299 cell lines.

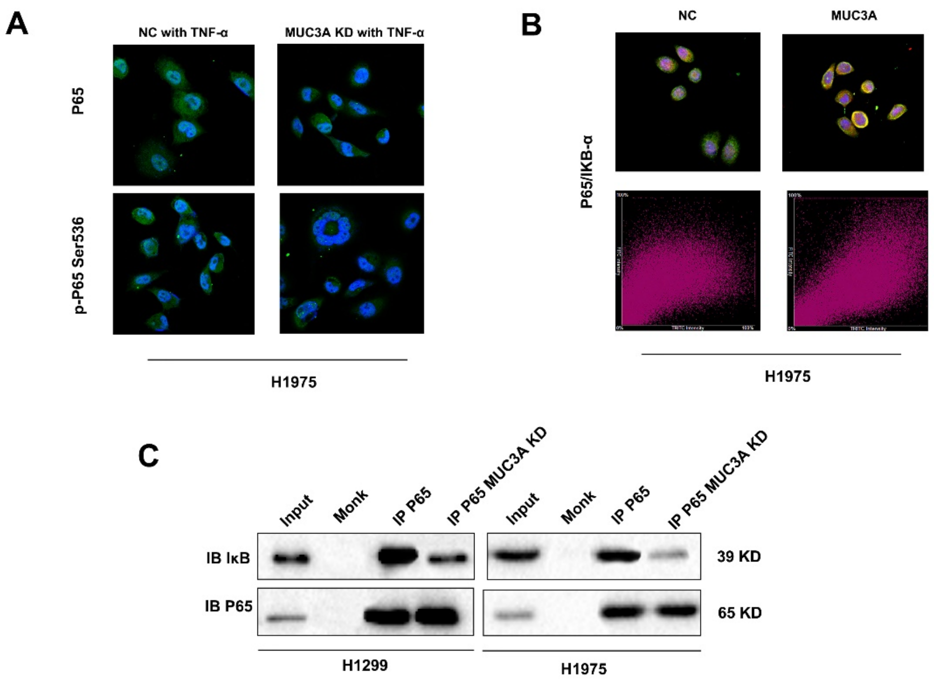

Figure S4. MUC3A interfered with the binding between P65 and IκB. (A) Representative images of p65 and p-p65 IF staining in H1975 cells. The MUC3A knockdown group had less p-p65 positive staining (green) than the control group. For total p65, there was no statistical difference between control and MUC3A knockdown groups. (B) IF to detect the binding condition of p65 (green) and IκB (red) in H1975. In the MUC3A knockdown cells, more p65 protein banded to IκB. (C) Co-IP to analyze the binding of p65 and IκB in control and MUC3A knockdown cells. In the MUC3A knockdown cells, the ratio of p65/IκB was significantly increased.

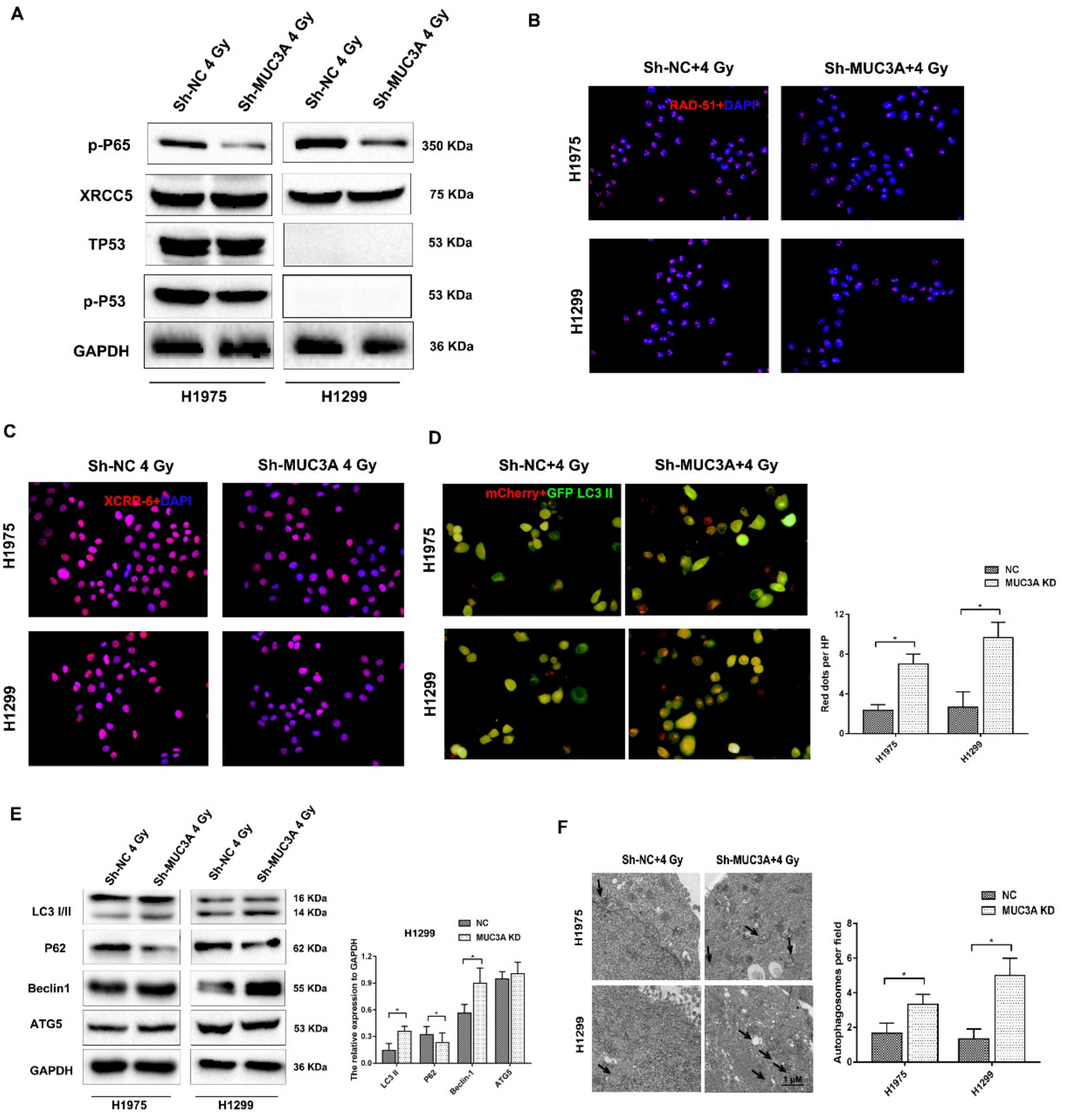

Figure S5. MUC3A facilitated the radiosensitivity of NSCLC cells. (A) Immunoblotting analysis of p-P65, XCRR-5, TP-53, and p-p53 protein levels in H1975 and H1299 parental and MUC3A knockdown cells after 4 Gy irradiation. (B) Representative images of RAD51 in H1975 and H1299 cells after 4 Gy irradiation. The MUC3A-knockdown group had fewer GADD45 foci (red) in the nuclear (blue) than the control group. *, $p < 0 .05$. (C) Representative images of XRCC 6 in H1975 and H1299 cells after 4 Gy irradiation. The MUC3A-knockdown group had fewer GADD45 foci (red) in the nuclear (blue) than the control group. *, $p < 0 .05$. (D) mCherry-GFP-LC3II adenovirus was infected into the cells for 24 hours and exposed to 4 Gy irradiation. MUC3A knockdown cells after 4 Gy irradiation-induced more red dots, indicating more late-stage autophagy. *, $p < 0 .05$. (E) TEM was applied to investigate the autophagy vacuoles formation. The black arrow point to an autophagy

vacuole. MUC3A knockdown cells with 4 Gy irradiation induced more autophagy vacuoles. *, $p < 0 .05$. (F) Immunoblotting analyses the autophagy relevant proteins, LC3, p62, Beclin1, and ATG5 in H1975 and H1299 parental and MUC3A knockdown cells. MUC3A-knockdown cells with 4 Gy irradiation promoted the expression of LC3II and Beclin-1 and decreased the level of p62. *, $p < 0 .05$.
